# A New Scoring System for Prediction of Intravenous Immunoglobulin Resistance of Kawasaki Disease in Infants Under 1-Year Old

**DOI:** 10.3389/fped.2019.00514

**Published:** 2019-12-11

**Authors:** Shu Wu, Yuan Long, Selena Chen, Yaqian Huang, Ying Liao, Yan Sun, Qingyou Zhang, Chunyu Zhang, Hui Yan, Jianguang Qi, Xueqin Liu, Yonghong Chen, Yong Zhang, Junbao Du

**Affiliations:** ^1^Department of Pediatrics, Peking University First Hospital, Beijing, China; ^2^Research Unit of Clinical Diagnosis and Treatment of Pediatric Syncope and Cardiovascular Diseases, Chinese Academy of Medical Sciences, Beijing, China; ^3^Department of Pediatric Cardiology, Wuhan Children's Hospital (Wuhan Maternal and Child Healthcare Hospital), Tongji Medical College, Huazhong University of Science and Technology, Wuhan, China; ^4^Division of Biological Sciences, University of California, San Diego, San Diego, CA, United States; ^5^Key Laboratory of Molecular Cardiovascular Sciences, Ministry of Education, Beijing, China

**Keywords:** Kawasaki disease, infants under 1-year old, intravenous immunoglobulin resistance, scoring system, prediction

## Abstract

**Background:** Children with Kawasaki disease (KD) under 1-year old are at high risk for intravenous immunoglobulin (IVIG) resistance. The study was designed to explore the predictive measure of IVIG resistance in infants under 1-year old with KD.

**Methods:** This study enrolled children under 1-year old suffering from KD in Peking University First Hospital and Wuhan Children's Hospital. All infants were divided into IVIG-responsive and IVIG-resistant groups. The differences in demographic characteristics, clinical features, and laboratory examinations were compared and the risk factors of IVIG resistant KD were analyzed. Furthermore, a scoring system was developed for predicting IVIG resistance in KD infants and an external validation was performed.

**Result:** A total of 282 infants (194 boys, median age of 7.0 months) were enrolled in this study, of whom 23 children were IVIG-resistant. Compared with IVIG-responsive infants, those in the IVIG-resistant group had a high neutrophil-to-lymphocyte ratio (NLR), high platelet-to-lymphocyte ratio (PLR), high mean platelet volume-to-lymphocyte ratio (MPVLR) in peripheral blood, and low serum albumin, and low serum sodium before IVIG therapy (all *P* < 0.01). Multiple regression analysis indicated that high levels of peripheral NLR and MPVLR, and low levels of serum albumin and serum sodium were independent risk factors for IVIG resistant KD infants. A scoring system, which included peripheral NLR ≥ 2.69 (1 point), MPVLR ≥ 2.78 (1 point), serum albumin ≤ 30.7 g/L (1 point), and serum sodium ≤ 135.2 mmol/L (1 point), was established. A cut-off value of a total score of 2 points or higher yielded a sensitivity of 87.0% and a specificity of 78.4%, with an area under the curve of 0.891. External validation with clinical diagnostic standard showed that a cut-off value of total score of 2 points or higher for predicting the IVIG-resistance yielded a sensitivity of 70.0% and a specificity of 75.1%.

**Conclusion:** For the first time, we proposed a predictive model of IVIG resistance in KD infants under 1-year old. The scoring system, which accounts for baseline peripheral NLR, MPVLR, and serum albumin and sodium, predicts with relatively high sensitivity and specificity for IVIG-resistant infants with KD under 1-year old.

## Introduction

Kawasaki disease (KD) commonly presents as an acute autoimmune vasculitis in childhood (1). Serious complications include coronary dilatation and coronary aneurysm, which may result in myocardial infarction (2, 3). Intravenous immunoglobulin (IVIG) with oral aspirin can significantly reduce the incidence of coronary artery complications (4). It is a standardized treatment for KD that is widely accepted (5). However, some children are resistant to IVIG therapy and have recurrent or persistent fever 36–48 h after the first dose of IVIG (4). The incidence of IVIG resistance was about 4.9–38.3% in different regions according to particular definition (4, 6–9). IVIG resistance represents severe inflammatory response and it is also an independent predictor for coronary artery lesions (10–12).

The peak incidence of IVIG resistance occurs at ages <1-year old, especially between 9 and 11 months old (9). Kobayashi et al. have shown that ages under 1-year old are an independent risk factor for not only IVIG resistance (13) but also coronary artery lesions (8). In recent years, randomized, open-label, blinded-endpoints trials have confirmed that IVIG therapy combined with other immunosuppressive agents such as glucocorticoid and cyclosporine effectively reduce the incidence of coronary artery complications in children predicted with IVIG resistance before treatment (14, 15). Therefore, it is important to determine an efficient predictive scoring system of IVIG resistance for KD infants under 1-year old.

Classic indicators previously identified to predict IVIG resistance include young ages, a high peripheral neutrophil percentage, high c-reactive protein, serum alanine transaminase (ALT), glutamyl transpeptidase, and total bilirubin levels, and low peripheral hemoglobin, serum albumin, and serum sodium levels (13, 16–21). Investigators have reported several scoring systems predicting IVIG resistance, for instance, the scoring systems by Sano, Kobayashi, and Egami scoring systems in Japan and San Diego scoring system in the United States. However, they showed unsatisfactory predictive abilities when validated externally in Chinese (22, 23). Recent studies showed that the neutrophil-to-lymphocyte ratio (NLR), platelet-to-lymphocyte ratio (PLR), and mean platelet volume-to-lymphocyte ratio (MPVLR) in peripheral blood could reflect the severity of inflammatory and cardiovascular disease (24–26). The basic pathological manifestation of KD is systemic vasculitis, and the increase of peripheral NLR and PLR are closely related to IVIG resistance (24, 27). However, at present the relationship between MPVLR and IVIG resistance remains unexplored. A previous study showed that in patients at all ages with KD, NLR ≥ 2.8 was a high risk factor for IVIG resistance (28), but the peripheral lymphocyte count or neutrophil count markedly changes with respect to the age groups in children. This has a significant influence on the predictive value of IVIG-resistant patients with KD.

Therefore, considering the specific impact of the peripheral lymphocyte count or neutrophil count according to age, and understanding that the peak incidence of IVIG resistance occurs at ages younger than 12 months old, the present study was undertaken to explore the predictive indicators of IVIG resistance to establish a Chinese scoring system predicting IVIG resistant KD infants under 1-year old.

## Materials and Methods

### Study Population

**Figure 1:**
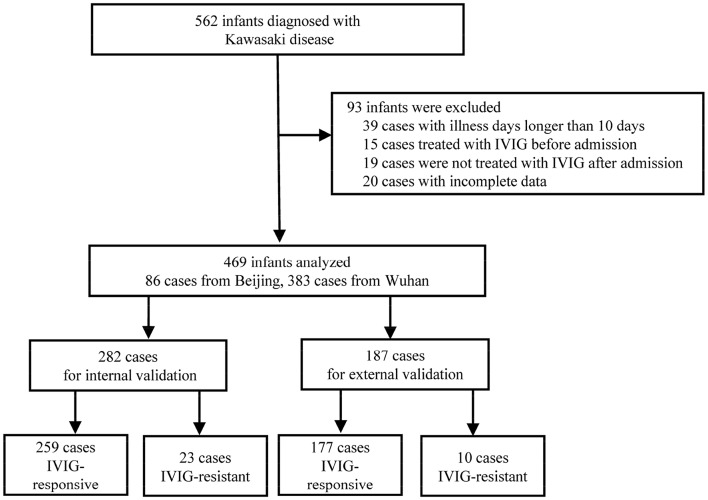
Flowchart of admitted patients. IVIG: Intravenous immunoglobulin resistance.

This research was a double-center-based retrospective study. The medical data of children under 1-year old diagnosed with KD in the department of pediatrics at Peking University First Hospital from January 2008 to August 2019 and Wuhan Children's Hospital from January 2018 to August 2019 were collected for constructing the predictive scoring system. Furthermore, the medical data of children under 1-year old diagnosed with KD in Wuhan Children's Hospital from January 2016 to December 2017 were used for external validation. All children met the KD diagnostic criteria by the American Academy of Pediatrics and the American Heart Association (29). The first day of illness was defined as the first day of fever. The following cases were excluded: (1) patients with illness days longer than 10 days; (2) patients treated with IVIG before admission; (3) patients without use of IVIG after admission; (4) patients with incomplete data (Figure 1). A total of 469 children were enrolled, receiving IVIG of 2 g/kg combined with oral aspirin of 30–50 mg/kg/d initially. IVIG resistance was defined as infants with KD having persistent or recrudescent fever (≥38°C) 48 h after completion of the first IVIG infusion (18). Two hundred eighty-two infants (259 IVIG-responsive cases and 23 IVIG-resistant cases) were used for the scoring system development to predict IVIG resistance in KD infants, and another 187 infants (177 IVIG-responsive cases and 10 IVIG-resistant cases) for the external validation (Figure 1). This study was approved by the Ethics Committee of Peking University First Hospital, China and the Ethics Committee of Wuhan Children's Hospital.

### Data Collection

Data referring to demographic characteristics, clinical manifestations, laboratory examinations before IVIG therapy, and echocardiography results were documented. The peripheral white blood cell count (WBC), neutrophil count, lymphocyte count, hemoglobin, platelet count, mean platelet volume, NLR, PLR, and MPVLR, together with ALT, albumin, and sodium in serum were recorded. We used echocardiography by two-dimensional ultrasound during hospitalization to assess coronary artery lesions. Coronary artery luminal diameters of the left main coronary artery and the right coronary artery were converted to body surface area-adjusted Z-scores. If the maximum Z-score of the coronary artery was >2.5, a coronary artery lesion was determined (29).

### Statistical Analysis

Statistical analysis was performed by SPSS version 25.0. We used frequency (percentage) to describe categorical variables and a χ^2^-test was used to analyze the difference between the 2 groups. For continuous variables, normally distributed variables were expressed as the mean ± standard deviation and assessed by independent sample *t*-test, and non-normally distributed variables were shown as median (interquartile range) and compared by the Mann-Whitney U test. Univariable analysis was performed to determine the differences in age (months), gender, peripheral WBC, hemoglobin, NLR, PLR, and MPVLR, and serum ALT, albumin, and sodium between two groups, and the continuous variables were converted to categorical variables first. Variables selected by the univariate analysis (*p* < 0.05) were applied for multivariate logistic regression to screen out independent risk factors for IVIG resistance. To construct the scoring system, the score of independent risk factors were determined by the odd ratios, and each patient obtained a total score. The cut-off point was chosen by the receiver-operator characteristic (ROC) curves and adjusted by the previous classical literature and clinical practice. The cut-off score was chosen at the highest Youden index and the sensitivity and specificity of the scoring system were analyzed. A value of *P* < 0.05 was considered statistically significant.

## Results

### Demographic and Clinical Features

One hundred ninety-four boys and 88 girls at a median age of 7.0 (4.0, 9.0) months were analyzed for establishing the scoring system in this study. There were 259 IVIG responders and 23 IVIG non-responders. IVIG resistance occurred in 8.16% of the study subjects. The IVIG-responsive group included 175 boys (67.6%) and 84 girls (32.4%) at a median age of 7.0 (4.0, 9.0) months. The IVIG-resistant group included 19 boys (82.6%) and 4 girls (17.4%) at a median age of 8.0 (5.0, 10.0) months. The percentage of patients with incomplete KD and coronary artery abnormalities between two groups did not differ (*P* > 0.05, Table 1). Compared with the IVIG-responsive group, the levels of peripheral neutrophil count, NLR, PLR, and MPVLR were significantly increased in IVIG-resistant patients, and the levels of peripheral lymphocyte and platelet count, serum albumin and sodium levels were significantly decreased (*P* < 0.01, except for the platelet count, *P* < 0.05; Table 1).

**Table 1:** Comparison of clinical characteristics between IVIG-responsive and IVIG-resistant cases.

**Variable**	**Total (*n* = 282)**	**IVIG-responsive (*n* = 259)**	**IVIG-resistant (*n* = 23)**	***P*-value**
Gender (M/F)	194/88	175/84	19/4	0.136
Age, months	7.00 (4.00, 9.00)	7.00 (4.00, 9.00)	8.00 (5.00, 10.00)	0.152
cKD/iKD	145/137	132/127	13/10	0.609
No. CAL	59 (20.9%)	53 (20.5%)	6 (26.1%)	0.525
White blood cell, 10^9^/L	14.55 (11.26, 18.85)	14.49 (11.20, 18.79)	14.71 (11.31, 22.63)	0.577
Neutrophil count, 10^9^/L	8.42 (5.91, 11.31)	8.13 (5.56, 11.14)	9.76 (8.14, 15.78)	0.009
Lymphocyte count, 10^9^/L	4.50 (3.06, 6.05)	4.63 (3.23, 6.16)	2.60 (1.88, 3.34)	<0.001
Hemoglobin, g/L	101.02 ± 11.93	101.10 ± 11.99	100.13 ± 11.48	0.709
Platelet count, 10^9^/L	402.00 (318.00, 513.00)	404.00 (324.00, 518.00)	324.00 (230.00, 484.00)	0.028
Mean platelet volume, fl	9.70 (9.10, 10.30)	9.70 (9.10, 10.30)	9.60 (9.30, 10.60)	0.628
NLR	1.93 (1.22, 2.90)	1.79 (1.20, 2.61)	4.86 (2.74, 6.26)	<0.001
PLR	94.59 (69.86, 125.14)	91.29 (69.55, 121.59)	132.74 (87.94, 198.08)	0.006
MPVLR	2.14 (1.57, 3.24)	2.07 (1.53, 3.00)	3.64 (2.78, 5.21)	<0.001
Alanine transaminase, IU/L	24.00 (15.00, 42.00)	23.00 (15.00, 40.00)	33.00 (23.00, 45.00)	0.057
Serum albumin, g/L	37.42 ± 4.42	37.68 ± 4.28	34.55 ± 5.03	0.001
Serum sodium, mmol/L	137.37 (135.50, 139.30)	137.60 (135.68, 139.40)	134.80 (133.21, 137.50)	0.001

*IVIG, intravenous immunoglobulin; cKD, complete Kawasaki disease; iKD, incomplete Kawasaki disease; No. CAL, numbers of coronary artery lesions; NLR, neutrophil-to-lymphocyte ratio; PLR, platelet-to-lymphocyte ratio; MPVLR, mean platelet volume-to-lymphocyte ratio*.

### Univariate Analysis

Ten categorical variables were analyzed in the univariate analysis. The cut-off point for each variable was as follows: (1) age ≤6 months; (2) gender, male; (3) peripheral WBC ≥ 14.5 × 10^9^/L; (4) peripheral hemoglobin ≤ 100.5 g/L; (5) peripheral NLR ≥ 2.69; (6) peripheral PLR ≥ 110.92; (7) peripheral MPVLR ≥ 2.78; (8) serum ALT ≥ 60 IU/L; (9) serum albumin ≤ 30.2 g/L; and (10) serum sodium ≤ 135.2 mmol/L. Peripheral NLR, PLR, and MPVLR, and serum albumin and sodium levels were significantly different between the two groups (*P* < 0.01, Table 2).

**Table 2:** Univariate analysis between IVIG-responsive and IVIG-resistant cases.

**Variable**	**Cut-off point**	**IVIG-responsive** **(*n* = 259)**	**IVIG-resistant** **(*n* = 23)**	**χ^2^**	***P*-value**
Age, months	≤6.0	158 (61.0%)	16 (69.6%)	0.655	0.418
Gender	Male	175 (67.6%)	19 (82.6%)	2.226	0.136
White blood cell, 10^9^/L	≥14.5	129 (49.8%)	12 (52.2%)	0.047	0.828
Hemoglobin, g/L	≤100.5	122 (47.1%)	11 (47.8%)	0.004	0.947
NLR	≥2.69	63 (24.3%)	18 (78.3%)	30.017	<0.001
PLR	≥110.92	83 (32.0%)	16 (69.6%)	13.052	<0.001
MPVLR	≥2.78	74 (28.6%)	19 (82.6%)	27.907	<0.001
Alanine transaminase, IU/L	≥40.0	64 (24.7%)	9 (39.1%)	2.289	0.130
Serum albumin, g/L	≤30.7	17 (6.6%)	9 (39.1%)	26.768	<0.001
Serum sodium, mmol/L	≤135.2	52 (20.1%)	13 (56.5%)	15.819	<0.001

*IVIG, intravenous immunoglobulin; NLR, neutrophil-to-lymphocyte ratio; PLR, platelet-to-lymphocyte ratio; MPVLR, mean platelet volume-to-lymphocyte ratio*.

### Multivariate Logistic Regression Analysis

Peripheral NLR, PLR, and MPVLR, and serum albumin and sodium were analyzed by multivariate logistic regression. The results indicated that peripheral NLR (≥ 2.69), MPVLR (≥ 2.78), serum albumin (≤ 30.7 g/L), and sodium (≤ 135.2 mmol/L) were independent risk factors for IVIG resistance with OR values of 4.027, 3.860, 3.300, and 3.700, respectively (Table 3).

**Table 3:** Independent factors identified by multiple logistic regression analysis for prediction of IVIG resistance.

**Variables**	**Logistic coefficient (β)**	**SE**	***Wald* χ^2^**	***P*-value**	**Odd ratio (95% CI)**	**Score point**
NLR ≥ 2.69	1.393	0.619	5.064	0.024	4.027 (1.197, 13.548)	1
PLR ≥ 110.92	0.551	0.594	0.860	0.354	1.735 (0.542, 5.557)	–
MPVLR ≥ 2.78	1.351	0.687	3.863	0.049	3.860 (1.004, 14.846)	1
Serum Albumin ≤ 30.7 g/L	1.194	0.600	3.961	0.047	3.300 (1.018, 10.693)	1
Serum Sodium ≤ 135.2 mmol/L	1.308	0.527	6.165	0.013	3.700 (1.317, 10.393)	1

*IVIG, intravenous immunoglobulin; CI, confidence interval; NLR, neutrophil-to-lymphocyte ratio; PLR, platelet-to-lymphocyte ratio; MPVLR, mean platelet volume-to-lymphocyte ratio*.

### Scoring System for Predicting IVIG Resistance

**Figure 2:**
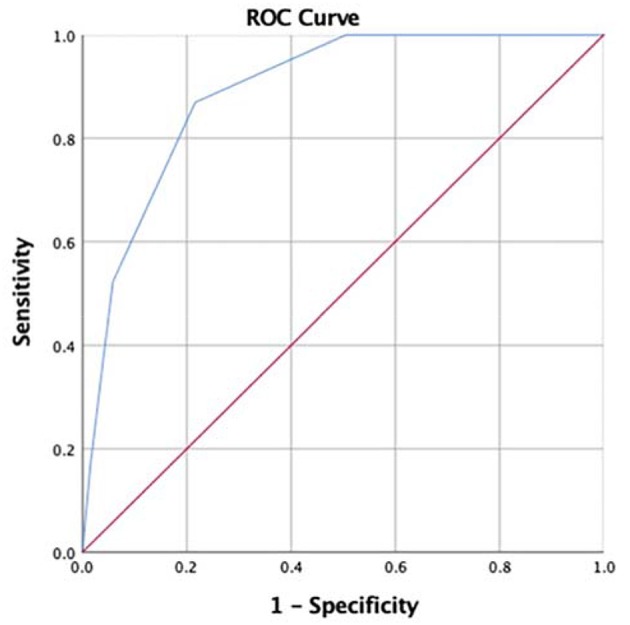
Receiver operating characteristic (ROC) curve of our scoring system for prediction of intravenous immunoglobulin (IVIG) resistance in Kawasaki disease (KD) patients under 1-year old. For the cut-off value of 2 points or more, the sensitivity and specificity were 87.0% and 78.4%, and the area under the curve (AUC) was 0.891 (95% confidence interval 0.837–0.945, *p* < 0.001).

To construct the predictive scoring system, peripheral NLR (≥2.69), MPVLR (≥2.78), serum albumin (≤30.7 g/L), and serum sodium (≤135.2 mmol/L) were all given 1 point depending upon the proximity of their odds ratio values. The total scores were calculated for each patient with KD. ROC analysis showed that the area under the curve (AUC) was 0.891 (95% confidence interval, 0.837–0.945; *P* < 0.001), and a cut-off score of 2 points or higher yielded the sensitivity of 87.0% and specificity of 78.4% to predict IVIG resistance (Figure 2).

### External Validation Studies

One hundred eighty-seven infants were enrolled in the externally validated population. External validation with clinical diagnostic standard showed that a cut-off value of total score of 2 points or higher for predicting the IVIG-resistance yielded a sensitivity of 70.0% and a specificity of 75.1% (Table 4).

**Table 4:** External validation of predictive scoring system.

**Prediction of IVIG resistance**		**Clinical standard-based outcome**, ***n*** **(%)**	
		**IVIG-resistant**	**IVIG-responsive**
Predictive scoring system-based outcome (≥2 points), *n* (%)	IVIG-resistant	7 (70%)	44 (24.9%)
	IVIG-responsive	3 (30%)	133 (75.1%)

*IVIG, intravenous immunoglobulin*.

## Discussion

Patients under 1-year of age diagnosed with KD are prone to be resistant to the initial IVIG treatment and develop coronary artery lesions. Our predictive model is the first scoring system for predicting IVIG-resistant patients with KD under 1-year old. The scoring system includes peripheral NLR ≥ 2.69 (1 point), peripheral MPVLR ≥ 2.78 (1 point), serum albumin ≤ 30.7 g/L (1 point) and serum sodium ≤ 135.2 mmol/L (1 point), and a total score ≥ 2 points yielded a sensitivity and a specificity of 87.0 and 78.4%, respectively, for predicting IVIG-resistance, and in external validation the sensitivity and specificity of predicting IVIG-resistance in KD infants were 70.0% and 75.1%, respectively.

The major pathological changes of KD were systemic vasculitis affecting small and medium-size arteries. Elevated peripheral NLR and MPVLR and decreased serum albumin and sodium represent the severity of inflammation. NLR stands for the ratio of absolute neutrophil count to lymphocyte count in peripheral blood. During systemic inflammation, increased neutrophil production in the bone marrow and circulation into blood, as well as delayed apoptosis, result in neutrophilia. Neutrophils play a critical role in the progression of vascular inflammation by migrating to the site of inflammation and releasing inflammatory cytokines and activating T cells. Meanwhile, accelerated apoptosis results from immunosuppression induced lymphocytopenia (30, 31). In consequence, a high level of peripheral NLR indicates the severity of the clinical course. Peripheral MPVLR represents the ratio of mean platelet volume to lymphocyte count, and high peripheral MPV values have been found in a variety of inflammatory diseases (32). Elevated MPVLR was shown in previous studies to predict the poor prognosis of patients with cardiovascular disease, especially for coronary heart disease (25, 33). This present study is the first to report that high MPVLR (≥2.78) is an independent risk indicator for predicting IVIG resistance in infants with KD under 1-year old.

The mechanisms of hypoalbuminemia consist of the following: first, increased vascular permeability leading to leakage of albumin (34, 35); second, liver dysfunction resulting in decreased albumin synthesis; and third, a lack of essential amino acids due to low nutrient intake or malnutrition, resulting in reduced albumin synthesis (36). IVIG non-responders tend to have more severe vascular leakage and liver damage, inducing lower albumin levels. The cause of hyponatremia is still unknown. Lim et al. found that there was a strong negative correlation between the level of serum sodium and inflammatory factors including c-reactive protein and interleukin-6 (IL-6) in children with KD (37). In addition to KD, studies referring to patients with inflammatory disease such as pneumonia, urinary tract infection, and lupus erythematosus also demonstrated that hyponatremia is an important marker for the severity and prognosis (38–40). The most probable pathophysiological mechanism for hyponatremia is non-osmotic secretion of antidiuretic hormone (ADH). Several studies have confirmed that the release of ADH is promoted by IL-6 and tumor necrosis factor-α (TNF-α) during inflammation (41). IL-6, TNF-α as well as other cytokines participate in inflammation of KD patients in the acute phase (42), suggesting that hyponatremia may be associated with inappropriate release of ADH. The marked increase in plasma IL-6 and TNF-α in IVIG-resistant infants compared with IVIG-responsive patients (43, 44) may explain the significant hyponatremia in IVIG non-responders. More serious inflammatory reactions at the acute phase in IVIG non-responders than in IVIG responders supports our findings that the inflammation-related indicators, including high peripheral NLR and MPVLR, and low serum albumin and sodium, could be used for predicting IVIG-resistant infants with KD under 1-year old effectively.

The indicators in our scoring system for predicting IVIG resistance, which include peripheral NLR and MPVLR and serum albumin and sodium, have significant advantages. They are inexpensive and easy-to-operate as routine examinations. Moreover, the peripheral neutrophil and lymphocyte are less influenced by age during the first 12 months. Our scoring system would have evident practical value for clinical applications due to its relatively high sensitivity and specificity.

There are some limitations to this study. The results may have bias as it was a retrospective study. The sample size was not large enough and a large-scaled external validation of our scoring system will be required in the future. However, the predictive model consisting of peripheral NLR (≥2.69) and MPVLR (≥2.78) and serum albumin (≤30.7 g/L) and sodium (≤135.2 mmol/L) prior to IVIG therapy showed relatively high sensitivity and specificity for the prediction of IVIG-resistant infants with KD under 1-year old.

## Data Availability Statement

The raw data supporting the conclusions of this article will be made available by the authors, without undue reservation, to any qualified researcher.

## Ethics Statement

The studies involving human participants were reviewed and approved by the Ethics Committee of Peking University First Hospital and the Ethics Committee of Wuhan Children's Hospital. Written informed consent for participation was not provided by the participants' legal guardians/next of kin because this is a retrospective study.

## Author Contributions

SW, YLo, YZ, and JD designed the study and analyzed the data. SW, YLo, YLi, YH, YS, CZ, HY, QZ, JQ, YC, XL, and YZ acquired the data. SW and YZ organized the database. SW, SC, and JD drafted the manuscript. YLo, YLi, YS, CZ, HY, QZ, JQ, YC, XL, YZ, and YH read and revised the manuscript. All authors contributed to all study data, write and approved the final version of the manuscript.

### Conflict of Interest

The authors declare that the research was conducted in the absence of any commercial or financial relationships that could be construed as a potential conflict of interest.
